# Evaluation of a deep-learning segmentation model for patients with colorectal cancer liver metastases (COALA) in the radiological workflow

**DOI:** 10.1186/s13244-025-01984-w

**Published:** 2025-05-23

**Authors:** Michiel Zeeuw, Jacqueline Bereska, Marius Strampel, Luuk Wagenaar, Boris Janssen, Henk Marquering, Ruby Kemna, Jan Hein van Waesberghe, Janneke van den Bergh, Irene Nota, Shira Moos, Yung Nio, Marnix Kop, Jakob Kist, Femke Struik, Nina Wesdorp, Jules Nelissen, Katinka Rus, Alexandra de Sitter, Jaap Stoker, Joost Huiskens, Inez Verpalen, Geert Kazemier

**Affiliations:** 1 Department of Surgery, Amsterdam UMC, Vrije Universiteit Amsterdam, Amsterdam, The Netherlands; 2 Cancer Center Amsterdam, Amsterdam, The Netherlands; 3 Department of Biomedical Engineering and Physics, Amsterdam UMC, University of Amsterdam, Amsterdam, The Netherlands; 4 Department of Radiology and Nuclear Medicine, Amsterdam UMC, University of Amsterdam, Amsterdam, The Netherlands; 5 Department of Radiology and Nuclear Medicine, Amsterdam UMC, Vrije Universiteit Amsterdam, Amsterdam, The Netherlands

**Keywords:** Colorectal neoplasms, Liver, Computed tomography, Artificial intelligence, Workflow

## Abstract

**Objectives:**

For patients with colorectal liver metastases (CRLM), total tumor volume (TTV) is prognostic. A deep-learning segmentation model for CRLM to assess TTV called COlorectal cAncer Liver metastases Assessment (COALA) has been developed. This study evaluated COALA’s performance and practical utility in the radiological picture archiving and communication system (PACS). A secondary aim was to provide lessons for future researchers on the implementation of artificial intelligence (AI) models.

**Methods:**

Patients discussed between January and December 2023 in a multidisciplinary meeting for CRLM were included. In those patients, CRLM was automatically segmented in portal-venous phase CT scans by COALA and integrated with PACS. Eight expert abdominal radiologists completed a questionnaire addressing segmentation accuracy and PACS integration. They were also asked to write down general remarks.

**Results:**

In total, 57 patients were evaluated. Of those patients, 112 contrast-enhanced portal-venous phase CT scans were analyzed. Of eight radiologists, six (75%) evaluated the model as user-friendly in their radiological workflow. Areas of improvement of the COALA model were the segmentation of small lesions, heterogeneous lesions, and lesions at the border of the liver with involvement of the diaphragm or heart. Key lessons for implementation were a multidisciplinary approach, a robust method prior to model development and organizing evaluation sessions with end-users early in the development phase.

**Conclusion:**

This study demonstrates that the deep-learning segmentation model for patients with CRLM (COALA) is user-friendly in the radiologist’s PACS. Future researchers striving for implementation should have a multidisciplinary approach, propose a robust methodology and involve end-users prior to model development.

**Critical relevance statement:**

Many segmentation models are being developed, but none of those models are evaluated in the (radiological) workflow or clinically implemented. Our model is implemented in the radiological work system, providing valuable lessons for researchers to achieve clinical implementation.

**Key Points:**

- Developed segmentation models should be implemented in the radiological workflow.
- Our implemented segmentation model provides valuable lessons for future researchers.
- If implemented in clinical practice, our model could allow for objective radiological evaluation.

**Graphical Abstract:**

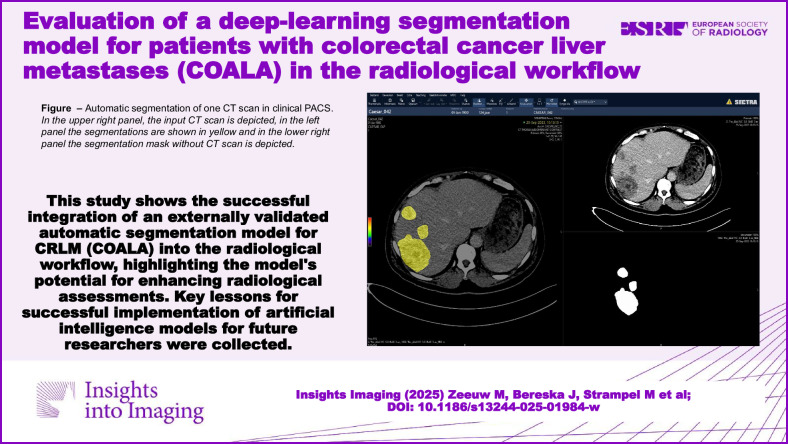

## Introduction

Total tumor volume (TTV) has been shown to be prognostic for survival outcomes in several solid tumors [1–4]. For patients with colorectal cancer liver metastases (CRLM), baseline TTV is prognostic for overall survival (OS), and change in TTV is prognostic for OS and recurrence-free survival (RFS) [5–7]. In conventional approaches, TTV is determined by manual segmentation of all tumors. However, manual segmentation is prone to inter- and intra-observer variability, and is a time-consuming and tedious task [8]. Consequently, using TTV as an imaging biomarker is not feasible in daily practice if measured manually.

Automatic segmentation models can potentially enhance reproducibility and facilitate clinical adoptability of volumetric tumor assessments in future daily practice. Moreover, currently used radiological assessments, such as the longest diameter used for evaluation of tumor response to systemic therapy, can be extracted from segmentations [9]. The adaptation of automatic segmentation models into clinical practice, however, necessitates a comprehensive evaluation of their performance, integration, and practical utility within the radiological workflow. For lung nodules, a computed tomography (CT)-based computer-aided system for detection, classification and growth rate estimation is already successfully implemented and clinically used in the radiological workflow in our tertiary center [10].

Recently, a deep-learning segmentation model for CRLM assessment called COALA (COlorectal cAncer Liver metastases Assessment) has been developed and externally validated [11]. Based on the tumor segmentations by COALA, automatic volumetric tumor assessment can be performed. During its development and validation, COALA was trained and tested in a research setting on patients with CRLM who participated in randomized clinical trials [12, 13]. To determine COALA’s clinical value and clinical utility, its performance should also be evaluated on patients in a clinical setting. Therefore, COALA was integrated with the picture archiving and communication system (PACS), a system used by radiologists for their daily clinical radiological assessments. Understanding the perspectives of end-users (e.g., radiologists) is essential for optimizing and refining COALA to meet the dynamic demands of clinical practice.

Automatic segmentation can lead to more consistent and objective radiological evaluations, thereby decreasing radiologists’ workload and facilitating clinical implementation and integration of TTV as an imaging biomarker for patients with CRLM. The evaluation of an automatic segmentation model in the radiological PACS is an essential step for ultimate implementation in daily practice.

The aim of this study is to evaluate the performance and practical utility of the deep-learning segmentation model COALA for patients with CRLM in the radiological workflow. A secondary aim is to provide valuable lessons for future researchers to implement artificial intelligence (AI) models.

## Methods

### Study population

This retrospective study was approved by the local medical ethics committee, and the requirement for informed consent was waived. Consecutive patients who received systemic therapy discussed at the CRLM multidisciplinary team meeting (MDT) between January and December 2023 were identified. All patients were discussed for the management of their CRLM with or without extrahepatic metastases. Patients with liver metastases from non-colorectal primary tumors were excluded. For patients to be included, contrast-enhanced portal-venous phase CT scans should be available in the PACS.

### End-users

Eight expert radiologists with 24 (Y.N.), 19 (J.H.T.M.W.), 11 (J.E.B.), 7 (M.P.M.K.), 5 (F.S.), 3 (J.W.K.), 3 (I.M.N.) and 2 (S.I.M.) years’ experience as abdominal radiologist were invited for an evaluation session. Of those, four were involved in the manual tumor segmentation of training data that was used for the development of COALA. For the evaluation, 90 min were scheduled at a clinical PACS workstation (IDS7, Sectra, Linköping, Sweden). This PACS is used by the radiologists for their daily clinical radiological assessments. Prior to the evaluation, instructions were provided to the radiologists on the procedure for accessing and viewing the segmentations in PACS. See the instructions in Supplement S1.

### Automatic tumor segmentation model COALA

A self-learning approach was followed to train the COALA segmentation model. Self-learning commences with a teacher segmentation model trained on a small set of manually segmented training data. This teacher segmentation model is then used to generate tumor segmentations for the entire unsegmented training dataset. These teacher-generated segmentations are subsequently used to train a student segmentation model. An nnUNet network setup that included a two-stage 3D U-Net cascade was selected for both the student and teacher segmentation models. The cascade comprised an initial U-Net trained on downsampled images to generate low-resolution segmentations, which served as an auxiliary input for training the subsequent full-resolution U-Net. A 5-fold cross-validation was used with an 80-20 training-validation split, 1000 steps per fold, and an initial learning rate of 0.05 to train both the low-resolution and full-resolution U-Nets. All models were trained on an NVIDIA A100 graphics processing unit (GPU), taking roughly one day per fold [11].

Input data for COALA were contrast-enhanced CT scans in the portal-venous phase. In total, 112 CT scans in axial view were retrospectively identified from the electronic health records (EHR). Subsequently, data were extracted from PACS, pseudonymized, and processed by COALA. Segmentation masks and reports with TTV were sent back to PACS. Scan acquisition parameters are summarized in Table 1.

**Table 1:** CT acquisition and reconstruction parameters

Parameter	CT scans *N* = 112
Table speed	69 (34–103) mm/s
X-Ray tube current	213 (152–336) mA
kVp	100 (100–100) kV
Tube rotation speed	35 (23–52) mm/rotation
Slice thickness	3 (3–3) mm
Width X height	512 × 512

Values are given as median (interquartile range)

### Implementation

**Fig. 1:**
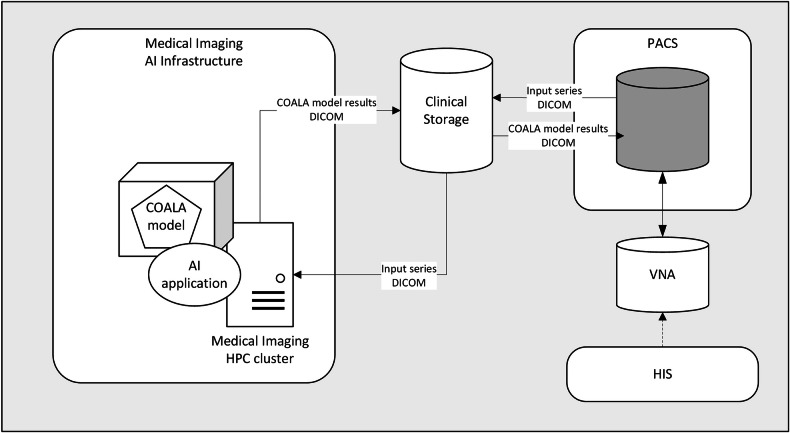
Data workflow: medical images are transported from PACS to HPC cluster via cache solution. AI, artificial intelligence; DICOM, digital Imaging and communications in medicine; HIS, health information system; HPC, high-performance computing; PACS, picture archive and communication system; VNA, vendor neutral archive

To create a stable and robust environment, COALA is packaged into a container, also containing a Python application with the necessary Python packages. The container is executed on a high-performance computing (HPC) cluster with specific GPU resources (NVIDIA P100—12GB). An application is built around the model to ensure that input and output data are according to the digital imaging and communications in medicine (DICOM) standard [14]. COALA performs the segmentations using GPU resources for processing speed. The input data was extracted from clinical PACS and stored in a cache. The cache functions as a bridge between the HPC cluster and the PACS. The segmentation results from the model (in DICOM) are sent back to the PACS via the same cache. Depending on the number of slices per CT scan, the automatic segmentation process varied from 25 to 40 min per CT scan. In Fig. 1, an overview of the data workflow is visible. For all DICOM transport, the Clinical Trials Processor (CTP) is used [15].

### Model evaluation

A questionnaire was developed for the evaluation of COALA in the radiological PACS system, composed together with abdominal radiologists, liver surgeons and medical imaging and ICT experts with experience in clinical implementation of in-house developed models. The questionnaire, following the 5-point Likert scale, served as a structured instrument to gather qualitative and quantitative feedback, facilitating a comprehensive analysis of the model’s strengths, weaknesses, and potential areas for further improvement. See Supplement S2 for the questionnaire. Upon the completion of all their assigned CT scan reviews, radiologists were asked to complete the questionnaire. Additionally, they were asked to provide any general observations or comments.

A qualitative analysis of the lessons learned during the development, validation and implementation phase of COALA was done by organizing feedback sessions with the end-users and research team during the development of COALA and after the evaluation sessions of the current study. During this feedback session, three key lessons were composed for future researchers implementing AI tools in the clinical workflow.

Categorical baseline variables were reported as numbers and percentages. Continuous baseline variables were reported as mean with standard deviation (SD) if normally distributed and as median with interquartile range (IQR) if not normally distributed. The distribution was analyzed using a histogram. IBM SPSS Statistics for Windows, version 28.0 (Armonk, NY: IBM Corp) was used.

## Results

### Imaging data

**Fig. 2:**
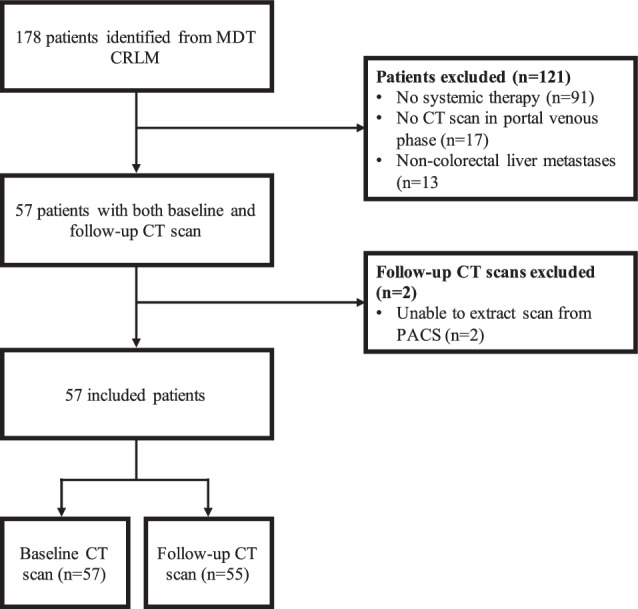
Patient inclusion flowchart. CRLM, colorectal cancer liver metastases; CT, computed tomography; MDT, multidisciplinary team; PACS, picture archiving and communication system

In total, 57 patients with a median age of 57 (IQR 53–72) years discussed at a CRLM MDT in a tertiary center were included in this study. Of those patients, 57 CT scans before systemic therapy and 55 CT scans after systemic therapy were available. See Fig. 2.

Less than half of the patients (42%) were female. The median number of CRLM at baseline was 3 (IQR 2–5) with a median largest diameter of 37 (IQR 22–55) mm. See Table 2.

**Table 2:** Patient baseline characteristics

Characteristics	Patients with CRLM *N* = 57
Female, *n* (%)	24 (42.1%)
Median age at baseline, years (IQR)	57 (53–72)
Median number CRLM at baseline (IQR)	3 (2–5)
Median largest diameter, mm (IQR)	37 (22–55)
CT scans before systemic therapy	57
CT scans after systemic therapy	55

*CRLM* colorectal cancer liver metastases, *CT* computed tomography, *IQR* interquartile range, *mm* millimeter

**Fig. 3:**
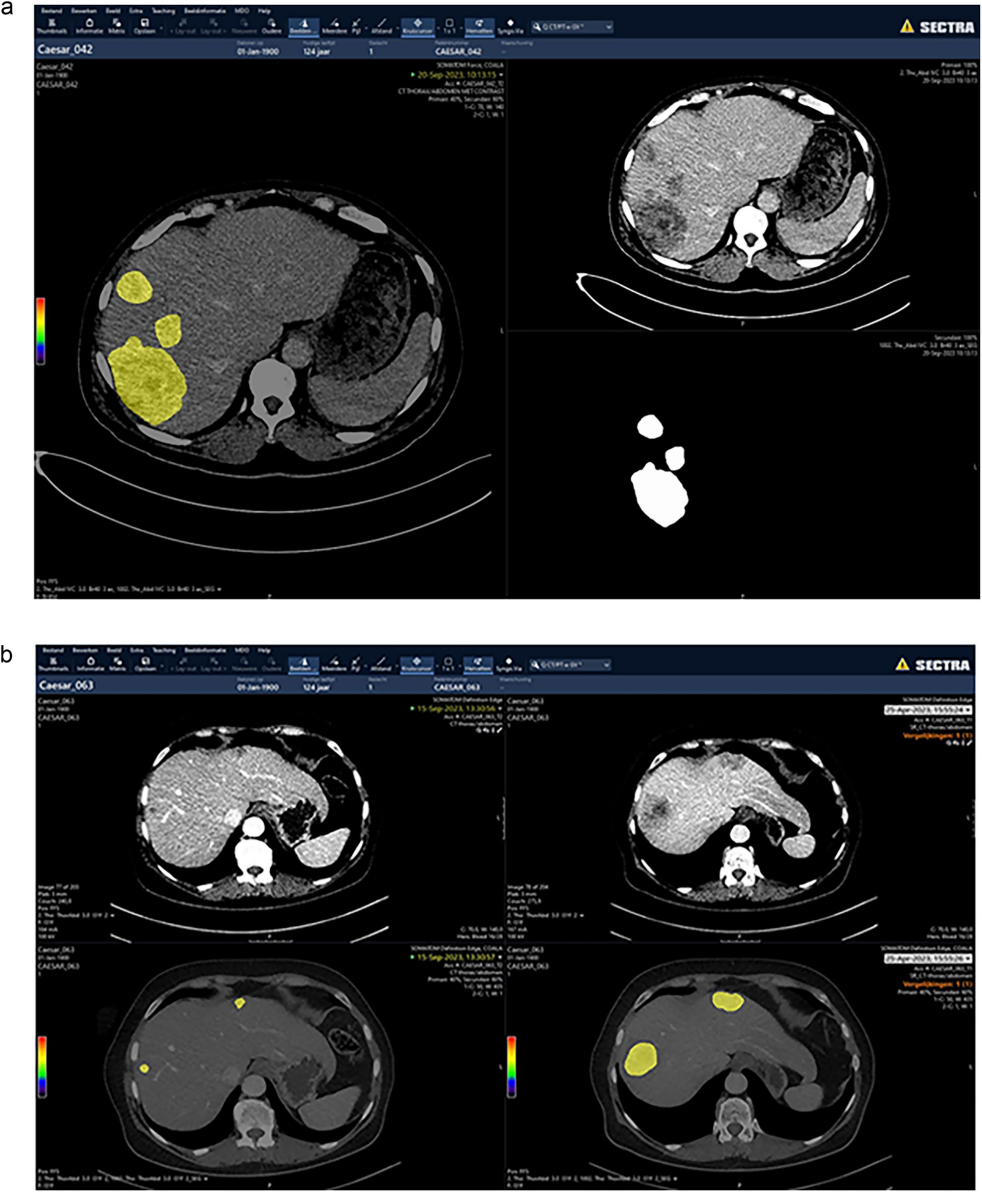
**a** Automatic segmentation of one CT scan in clinical PACS. In the upper right panel, the input CT scan is depicted, in the left panel, the segmentations are shown in yellow, and in the lower right panel, the segmentation mask without CT scan is depicted. **b** Two CT scans before and after systemic therapy with automatic segmentation in clinical PACS. In the left panel, the input CT scan without (upper panel) and with (lower panel) segmentation is shown after systemic therapy. In the right panel, the input CT scan without (upper panel) and with (lower panel) segmentation is shown before systemic therapy. CT, computed tomography; PACS, picture archiving and communication system

Figure 3a displays an example of a single CT scan with an automatic segmentation in the PACS. Concurrently, Fig. 3b presents a comparative analysis of two CT scans of the same patient: one before systemic therapy and one after systemic therapy, each augmented with automatic segmentation.

### Assessment of the performance and clinical integration

**Fig. 4:**
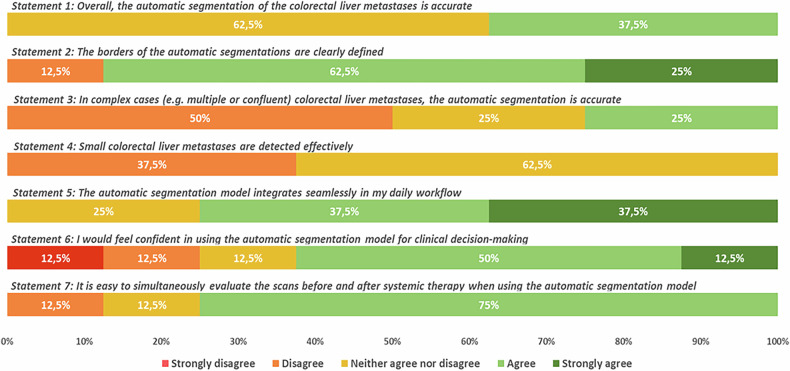
The answers to the questionnaire of the eight expert abdominal radiologists

Figure 4 presents the feedback from the eight radiologists after assessing 14 CT scans each. The results showed that the overall segmentation was deemed accurate by 3 (37.5%) radiologists (‘’agree”), with 5 (62.5%) radiologists concurring (“agree”) that the segmentation borders were distinctly outlined, and 2 (25%) expressing strong agreement. However, the agreement diverged for more intricate scenarios, with a 50% disagreement rate for complex (e.g., confluent or heterogeneous lesions) cases and a 37.5% disagreement rate for cases involving small CRLM.

Six of the eight participating expert radiologists reported that the model seamlessly integrated into their clinical workflow, with 37.5% agreeing and an equal percentage strongly agreeing. The majority (6 radiologists, 75%) agreed that assessing scans both before and after systemic treatment was not hindered when using the model’s outputs. However, their confidence in the model’s findings to be used for clinical decision-making exhibited a broad spectrum of responses: 1 (12.5%) strongly disagreed, 1 (12.5%) disagreed, 1 (12.5%) remained neutral, 4 (50%) agreed, and 1 (12.5%) strongly agreed.

The free text feedback showed that, while the detection rate for CRLM was high, a recurring observation was the underestimation of lesion size, particularly in smaller CRLM. Additionally, in cases of heterogeneous CRLM, such as those with calcifications, segmentation was limited to the hypodense regions, omitting the hyperdense areas. Subcapsular lesions, especially those near the diaphragm or heart, were frequently not detected.

### Lessons learned

Three key lessons were identified during the development, validation and implementation process, which we deem useful for future researchers. The first was a multidisciplinary collaboration, involving clinical and technical researchers, the envisioned end-users, clinicians treating patients with CRLM, and medical imaging & ICT experts with experience in clinical implementation of in-house developed models. The second was proposing a robust methodology prior to developing and externally validating the model. This assures the generalizability of the model to unseen datasets. Lastly, by involving the end-users early in the development phase, their insights were gathered prior to development, allowing it to be fine-tuned to their needs and demands. Moreover, by organizing evaluation sessions with expert abdominal radiologists, valuable insights were gathered for further optimization and integration of the model in the clinical setting.

## Discussion

This study demonstrates that the deep-learning segmentation model COALA for patients with CRLM is user-friendly when used within the radiological PACS. The evaluation sessions with expert abdominal radiologists provided valuable insights for further optimization and integration of COALA in the clinical setting, such as the decreased segmentation performance in smaller lesions, heterogeneous lesions and lesions at the border of the liver. Key lessons for successful implementation of AI models for future researchers were a multidisciplinary approach, proposing a robust method prior to model development with external validation and organizing feedback sessions with the end-users early in the development phase of the model, to meet their demands and needs.

To facilitate the clinical implementation of automatic segmentation models, seamless integration in the daily workflow of clinical radiologists is crucial. By involving the end-user (i.e., abdominal radiologists) in the development process and testing the model in their daily workflow, essential improvements can be made. Some radiologists evaluated the performance of the model as moderate, but did report that they would be confident in using the model for clinical decision-making. The reason for this is that, especially in small lesions, the performance was reported to be lower. In large lesions, however, the performance was deemed accurate by most radiologists. In current practice, only the two largest CRLM are used for RECIST1.1 assessment, explaining the seemingly contradictory outcome in evaluated model performance and confidence in clinical decision-making. The possibility of automating manual radiological evaluations, such as response evaluation using RECIST1.1 criteria through automated measurements, stands out as a promising application. Additionally, the model has the potential to advance TTV as an imaging biomarker in clinical practice, currently hindered by a labor-intensive manual segmentation process.

A strength of this study is that the patients included for this clinical evaluation resembled the clinical practice better than the patients used for model development. The latter group consisted of patients enrolled in an RCT, all meeting highly specific inclusion criteria to be enrolled in the trial. Patients from the CAIRO5 trial suffered from initially unresectable liver-only CRLM and had never undergone local treatment (surgery and/or ablation) before. Patients in this study were randomly identified from the CRLM MDT, some of them suffering from recurrent CRLM after local treatment, resulting in livers with operation clips and/or post-ablation zones. Testing COALA on such patients ascertains the generalizability of the model by gaining insights into how to optimize COALA’s performance on patients with small lesions and previously treated livers.

Various other segmentation models have been developed for patients with CRLM. None of the earlier developed models were, however, evaluated in the radiological workflow or clinically implemented. Most of the models never surpass the development phase and are only internally validated [16–18]. One group did externally validate their automatic segmentation model, resulting in a significant decrease in performance in the external cohort [19]. These findings are in concordance with findings by other research groups focusing on the clinical implementation of models. This emphasizes the critical need for external validation and clinical evaluation to ultimately facilitate clinical implementation [20, 21].

This study has several limitations, such as the ongoing development of radiological features like automatic tumor diameter measurements and lesion tracking over time. Both the model and the application need to be further developed to include these features. A more seamless integration in the hospital IT infrastructure is possible with the use of further containerization, streaming, and by deploying a web-API. Overall, the robustness of the software code could increase with the use of automatic deployments. Furthermore, the model is integrated into the PACS of the current vendor of our academic hospital. Adaptation to a different PACS could potentially pose challenges, although the produced results are DICOM conform. Lastly, an impediment to the complete integration of the model in the radiological workflow is the absence of a clear triggering system for the model. At the moment, communication between EHR, Radiology Information System, PACS and HPC cluster is fragmented, and therefore, a manual trigger to run the model for a specific patient remains necessary. Several institutes have taken effort to describe and build such a dedicated AI deployment infrastructure [22]. Standards-based guidelines for AI integration developed by Integrating the Healthcare Enterprise (IHE) Radiology AI Workflow are currently in trial implementation [23].

Future directions include a thorough assessment of the model’s segmentation and volumetric assessment capabilities during MDTs, specifically focusing on its ease of use, interpretability, and, foremost, clinical relevance.

In summary, this study demonstrates the successful integration of an externally validated automatic segmentation model for CRLM (COALA) into the radiological PACS, highlighting the model’s potential for enhancing radiological assessments. Valuable insights for further optimization and integration of COALA in the clinical setting were gathered. Key lessons for successful implementation for future researchers were a multidisciplinary approach, proposing a robust method prior to model development and organizing feedback sessions with the end-users early in the development.

## Supplementary information

ELECTRONIC SUPPLEMENTARY MATERIAL

## Data Availability

The authors confirm that the data supporting the findings of this study are available within the article and its supplementary material. The (imaging) data can be obtained by researchers upon request.

## Abbreviations

AI: Artificial intelligence
CRLM: Colorectal cancer liver metastasis
COALA: COlorectal CAncer Liver metastasis Assessment
CT: Computed tomography
DICOM: Digital imaging and communications in medicine
EHR: Electronic health records
GPU: Graphic processing unit
HPC: High performance computing
MDT: Multidisciplinary team meeting
PACS: Picture archiving and communication system
TTV: Total tumor volume

## Code availability

The documented code, fully trained COALA model, is available on GitHub.
