# Supplementary material for: Evaluation of a deep-learning segmentation model for patients with colorectal cancer liver metastases (COALA) in the radiological workflow
Source: Insights Imaging. 2025 May 23;16:110. doi: 10.1186/s13244-025-01984-w (PMC12102431; doi:10.1186/s13244-025-01984-w)
Supplement: Supplementary file 1 — ELECTRONIC SUPPLEMENTARY MATERIAL [file 13244_2025_1984_MOESM1_ESM.pdf]

Evaluation of a deep-learning segmentation model for patients with colorectal cancer liver metastases (COALA) in the radiological workflow

ELECTRONIC SUPPLEMENTARY MATERIAL

Supplement S1 – Instruction for accessing and viewing the segmentation in PACS

Step 1: Read all the questions of the questionnaire carefully.

Step 2: Find the study patient in PACS, and open CT scan(s).

Step 3: Click right mouse, choose 'Overlay' and subsequently choose the segmentation mask next to the corresponding CT scan.

Step 4: By double clicking both left and right mouse, the opacity of the segmentation mask can be adjusted.

Step 5: Assess the scan(s) as if it is a clinical patient, and assess accuracy of segmentation.

Step 6: Fill out the questionnaire.

Supplement S2 – Questionnaire

1. Overall, the automatic segmentation of the colorectal liver metastases is accurate
   1) Strongly disagree
   2) Disagree
   3) Neither agree nor disagree
   4) Agree
   5) Strongly agree

2. The borders of the automatic segmentations are clearly defined
   1) Strongly disagree
   2) Disagree
   3) Neither agree nor disagree
   4) Agree
   5) Strongly agree

3. In complex cases (e.g. multiple or confluent) colorectal liver metastases, the automatic segmentation is accurate
   1) Strongly disagree
   2) Disagree
   3) Neither agree nor disagree
   4) Agree
   5) Strongly agree

4. Small colorectal liver metastases are detected effectively
   1) Strongly disagree
   2) Disagree
   3) Neither agree nor disagree
   4) Agree
   5) Strongly agree

5. The automatic segmentation model integrates seamlessly in my daily workflow
   1) Strongly disagree
   2) Disagree
   3) Neither agree nor disagree
   4) Agree
   5) Strongly agree

6. I would feel confident in using the automatic segmentation model for clinical decision-making
   1) Strongly disagree
   2) Disagree
   3) Neither agree nor disagree
   4) Agree
   5) Strongly agree

7. It is easy to simultaneously evaluate the scans before and after systemic therapy when using the automatic segmentation model
   1) Strongly disagree
   2) Disagree
   3) Neither agree nor disagree
   4) Agree
   5) Strongly agree

8. Open field (for general remarks)
